# Novel Bending Test Method for Polymer Railway Sleeper Materials

**DOI:** 10.3390/polym13091359

**Published:** 2021-04-21

**Authors:** Choman Salih, Allan Manalo, Wahid Ferdous, Rajab Abousnina, Peng Yu, Tom Heyer, Peter Schubel

**Affiliations:** 1 Centre for Future Materials (CFM), Faculty of Health, Engineering and Sciences, University of Southern Queensland, Toowoomba, QLD 4350, Australia; Choman.Salih@usq.edu.au (C.S.); Allan.Manalo@usq.edu.au (A.M.); Peng.Yu@usq.edu.au (P.Y.); Peter.Schubel@usq.edu.au (P.S.); 2 School of Engineering, Macquarie University, Macquarie Park, NSW 2113, Australia; rajab.abousnina@mq.edu.au; 3 Austrak Pty Ltd., Brisbane, QLD 4000, Australia; Tom.Heyer@vossloh.com

**Keywords:** timber replacement sleeper, composite sleeper, five-point bending test, Beam on Elastic Foundation (BOEF), in-track sleeper behaviour

## Abstract

Alternative sleeper technologies have been developed to address the significant need for the replacement of deteriorating timber railway sleepers. The review of the literature indicates that the railway sleepers might fail while in service, despite passing the evaluation tests of the current composite sleeper standards which indicated that these tests do not represent in situ sleeper on ballast. In this research, a new five-point bending test is developed to evaluate the flexural behaviour of timber replacement sleeper technologies supported by ballast. Due to the simplicity, acceptance level of evaluation accuracy and the lack of in-service behaviour of alternative sleepers, this new testing method is justified with the bending behaviour according to the Beam on Elastic Foundation theory. Three timber replacement sleeper technologies—plastic, synthetic composites and low-profile prestressed concrete sleepers in addition to timber sleepers—were tested under service loading condition to evaluate the suitability of the new test method. To address the differences in the bending of the sleepers due to their different modulus of elasticities, the most appropriate material for the middle support was also determined. Analytical equations of the bending moments with and without middle support settlement were also developed. The results showed that the five-point static bending test could induce the positive and negative bending moments experienced by railway sleepers under a train wheel load. It was also found that with the proposed testing spans, steel-EPDM rubber is the most suitable configuration for low bending modulus sleepers such as plastic, steel-neoprene for medium modulus polymer sleepers and steel-steel for very high modulus sleepers such as concrete. Finally, the proposed bending moment equations can precisely predict the flexural behaviour of alternative sleepers under the five-point bending test.

## 1. Introduction

Several composite sleeper technologies have been introduced as alternatives to address the issue of environmental deterioration and scarcity of hardwood timber sleepers. Even 0.5% of in-service traditional prestressed concrete sleepers fail and require replacement annually, while nearly 1% are discarded due to defects in the manufacturing [1]. The main alternative sleepers are synthetic composites (SC) [2] recycled plastics [3,4], low-profile prestressed concrete [5], polymer concrete [6] and steel sleepers [7]. Due to their superior durability, strength/weight ratio, environmental-friendly properties, and excellent resistance to rot and insect attack, alternative sleeper materials from fibre composites and recycled plastics gained significant attention [8,9,10,11]. It is estimated that the market share of composites sleepers in the passenger rail will increase to 4.6% in 2024 due to the ever-increasing demand for lightweight materials with exceptional mechanical properties [12]. For example, Queensland Rail in Australia needs at least 100,000 composite sleepers per year in the next 5 years to ensure the continuous operation of its regional rail network [13]. While these alternative sleeper technologies have been available for more than two decades, their use in actual railway tracks is still marginal as compared to timber, concrete and steel sleepers [14]. Qiu et al. [15] related this low usage of composite sleepers to the lack of recognised unified national and international standards for alternative sleeper materials. For example, the AREMA standard specifies the minimum modulus of elasticity of alternative sleepers to not be less than 1.17 GPa [16], while the Japanese standard (JIS 1203:2007) specifies a minimum of 6 GPa [17]. This requirement according to ISO 12856-1:2014 ranges from 1.17 to 6 Gpa [18], while the recently developed Australian Standard AS1085.22:2020 does not specify any value of modulus of elasticity for an alternative sleeper [19]. Moreover, the evaluation tests according to these standards are mostly based on prestressed concrete sleepers, which does not represent exactly the behaviour of alternative sleeper materials supported on a ballast, as reviewed in Section 2.

**Figure 1:**
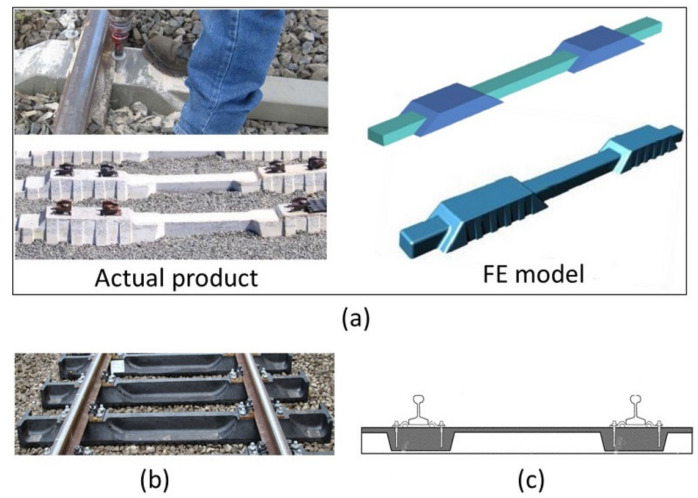
Different shapes of alternative sleepers: (**a**) composite sleepers [28,29,30], (**b**) recycled plastic sleeper [31] and (**c**) alternative composite sleeper patent [32].

Railway sleepers are loaded with two wheels on the rail seat areas, which produces positive bending moments on these areas while simultaneously produce negative bending moment at the centre section. Past research indicates that the stress distribution of the rail seat area is significantly higher than that of the centre part [20,21]. It is believed that the AREMA standard overestimates the flexural requirement of concrete sleepers for the rail seat area [22] but not the centre part, as it has been reported that centre cracking is one of the most common failure types of concrete sleepers [23,24]. In-service evaluation of the flexural capacity of concrete sleepers showed higher capacity than that of the AREMA requirement, yet centre cracking was observed [22]. A similar situation can be seen for plastic composite sleepers where the in-service monitoring of these sleeper types indicated centre cracking failure within the first 15 years of their installations [25]. Plastic sleepers are, however, designed to provide an average service life of ~40 years [26]. This indicates that the estimations and test evaluation tests according to the existing standards may not represent the loading condition of sleepers supported by track. This issue was highlighted by McHenry et al. [25], wherein they indicated that the static flexural tests in the AREMA standard were originally designed to simulate the high bending stresses induced during installation, which is a one-off phenomenon. Moreover, a recent study on the hogging bending deflection behaviour of sleepers showed that none of the standard tests completely represent the in-service behaviours [27]. Therefore, a new five-point bending test is proposed in this research aiming to induce realistic in situ bending moments at the rail seat and centre of the sleepers simultaneously. Not only is this research beneficial in the evaluation of the existing composite sleepers, but also in the evaluation of futuristic composite sleepers with different sectional properties due to shape and material optimisations (Figure 1). The latter is important because full-size sleepers will undergo positive and negative bending (known as W-shape), and thus any unexpected failure such as design integrity and materials interface failure might be captured by this type of test. Moreover, as the review of the existing standards in Section 2 indicated, several sleeper sections need to be tested to evaluate sleepers’ positive and negative bending moments while in the five-point bending one test can evaluate both bending behaviours.

This paper presents the development of the five-point bending test for composite sleepers, and its suitability is demonstrated through full-size static test under service load. First, an analysis of different composite sleepers supported by ballast was conducted using Strand 7 software to understand the bending moment intensity and its variation from the rail seat to the centre of a sleeper and the change to another sleeper type. The full sleeper support condition was assumed as it was shown that the deflection of partially supported sleepers are not very much different (within 5–8%) to that of the full supported sleepers [33]. The Beam on Elastic Foundation theory (Winkler model) was used to validate the computer model due to its reasonable estimation accuracy [20,34] and lack of in-service bending behaviour of various sleepers types. Second, the testing spans were adjusted so that similar bending moments to that of the realistic conditions are induced. Further, to prevent high shear (and possibly shear failure) and to consider the change in the positive-to-negative bending moment ratio from a sleeper type to another, different elastic support types were considered, and the best one is selected for specific material type. The Digital Image Correlation (DIC) technique was employed to capture the bending behaviour through full-profile deflection shapes and the support settlement measurements. Finally, analytical solutions of the five-point bending test based on the classical beam theory were developed and validated with Finite Element Analysis (FEA) to calculate the magnitude of bending moment at the rail seat and centre of an alternative sleeper.

## 2. Evaluation of the Existing Test Standards 

The flexural behaviour evaluation of alternative sleeper technologies is a fundamental part of understanding sleeper’s mechanical properties. It is also one of the required tests for the performance evaluation of a newly developed sleeper design for their acceptance in real-world applications. However, it has been demonstrated that the in-track flexural behaviour of sleepers with significant differences in their elastic moduli, *E_s_*, are different. For example, the composite sleepers (*E_s_* = 8 GPa) will have a higher deflection when compared to a prestressed concrete sleeper (*E_s_* = 36 GPa) [35], and this is also different from a typical hardwood timber [36]. This has long-term negative effects on the performance of the tracks as stiffer sleepers will attract more loads while lesser stiff sleepers will carry a very minimal load when they are interspersed with timber sleepers [18,19]. Therefore, accurate evaluation of the flexural behaviour of alternative sleeper materials is essential to manage the tracks appropriately. Accordingly, a few test standards [16,17,18,19] have been published in the last two decades to help facilitate the design and manufacture of alternative sleeper materials. The test methods for evaluating the performance of sleepers suggested in these standards are presented and evaluated in this section. 

The bending tests are requirements for all of the existing standards to demonstrate the suitability of an alternative sleeper technology. As shown in Table 1, these tests are of three-point or four-point bending configurations usually with resilient pads under the loading point and over the supports. Table 1 highlights the main differences among the different standards, focusing on the bending tests.

The AREMA standard [16] highlighted that the full test criteria have not been developed for alternative sleepers. Therefore, researchers need to take this into account and be more cautious when designing and evaluating an alternative sleeper according to the relevant standard. For example, material characterisation according to the new Australian standard AS1085.22 [19] is recommended to be carried out based on ISO 12856-1 [18], which was specifically developed for plastic sleepers. However, AS1085.22 [19] is intended to cover all alternative sleeper materials that are not timber, concrete or steel. The same can be seen in the bending test configurations of the AS1085.22 [19] standard for alternative sleepers that are identical to the tests in the AS1085.14 [37] and AREMA-chapter 30-4 [16], which are developed for prestressed concrete sleepers. The tests in both ISO 12856-2 [38] and AREMA-chapter 30-5 [16] can be followed in the evaluation of plastic sleepers as both cover plastic sleeper requirements; however, the ISO standard allows the sleepers to be tested with rail pads attached to them, while the AREMA does not. Another major difference between the standards is in their test/passing criteria. AREMA [16] does not specify any pass criteria for alternative sleepers; however, prestressed concrete sleepers shall not develop structural cracks when tested according to Section 4 of the standard. On the other hand, the Australian AS1085.22 [19] states that the permanent deflection should be less than 0.5 mm after three minutes from unloading in addition to the no crack requirements. 

**Figure 2:**
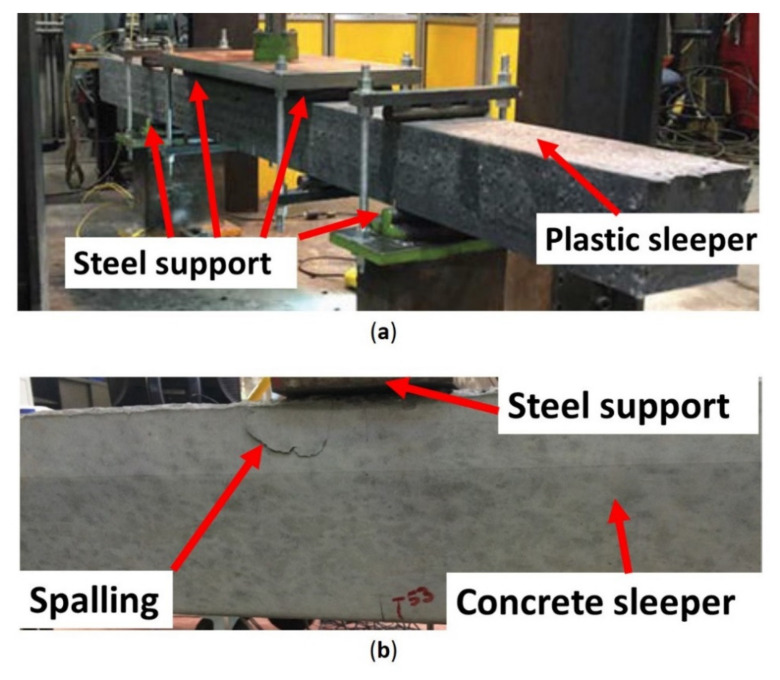
(**a**) Four-point bending test of a plastic sleeper without resilient pads [25]. (**b**) Spalling of the concrete surface under a steel plate [39].

The bending tests according to these standards have limitations in regards to how a sleeper on ballast behaves according to BOEF theory. First, these standards evaluate the bending behaviour in two to four separate tests which are not only time and resource-consuming but also do not represent accurately the behaviour of a sleeper on ballast where the sleepers are experiencing both negative and positive bending moments. Second, the available test standards do not consider a rail section or similar-sized steel plates for the application of loads on the rail seats. This can be critical in evaluating if the sleepers will experience any indentation in the rail seat area, especially for alternative designs that omit the use of rail pads. Moreover, the use of resilient pads in the standards may not have a practical reason that reflects the sleeper on ballast behaviour or the necessity according to material characteristics. For example, it might be necessary to use resilient pads for concrete sleepers (as required by AREMA-chapter 30-4 and AS1085.14 standards) to prevent spalling of concrete and hence preventing cross-sectional loss as shown in Figure 2b, where the load was applied directly onto the concrete surface [39]. It seems that this requirement is carried over to the new alternative standards, according to which the sleepers have much lower moduli of elasticity than that of concrete and thus may not require these elastic pads. A recent study on the improvement of the testing method for composite sleepers (plastic sleepers tested) based on the AREMA [16] considers a four-point bending test without any resilient pads, as shown in Figure 2a [25]. In the present study, however, elastic pads were used to redistribute the bending moments according to BOEF theory (accounting for the sleepers’ modulus of elasticity), thus mimicking and capturing the behaviour of alternative sleepers on ballast. 

While it is evident that a sleeper in-track is subjected to both positive and negative bending moments at the rail seat and the centre, the intensity of bending moments at the rail seat and centre are different from a sleeper to another because of changes in the stiffness of the sleepers. Therefore, a new test method is proposed with a view of representing the actual behaviour of railway sleepers over the ballast. 

## 3. The Concept of Five-Point Static Bending for Railway Sleepers

**Figure 3:**
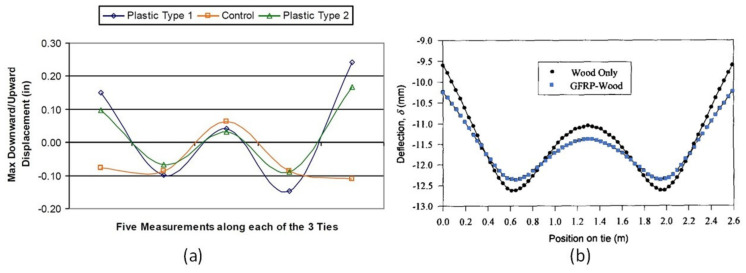
(**a**) In−service bending shape of plastic and timber sleepers [43]. (**b**) Deflection profile of timber with and without reinforcement according to BOEF [44].

As railway sleepers are supported by ballast, the rail seats are under a positive bending moment (sagging moment) while the centre of the sleeper is under a negative bending moment (hogging moment). The design of railway sleepers has been based on the BOEF theory which was first introduced by Winkler in 1867 and Zimmermann in 1888 [40] and later modified by Heteneyi in 1967 [41]. Due to the lack of comprehensive data on the in-tack bending behaviour of composite sleepers with various bending modulus in Australia, this method is used for the estimation of bending behaviour of the sleeper samples with acceptable accuracy as highlighted by Zakeri and Sadeghi (2007) [20]. This model was also introduced in the AS1085.14:2012 [37] as an alternative to the empirical method of sleeper analysis. The limitations of the BOEF model, however, should be noted, such as the lack of the interaction between the supporting layers of the sleeper (ballast, sub-ballast and subgrade) because the model only represents these layers with one support modulus value [42]. According to the BOEF model, the intensity of the bending moments in both the rail seat and the sleeper centre is affected by the condition of the ballast (sleeper support modulus), gauge width/sleeper length and the sleeper material type. This indicates that a composite sleeper with an elastic modulus of ~2 GPa will have a different bending behaviour to that of a 40 GPa prestressed concrete sleeper supported by ballast. The general bending shape of a loaded sleeper in-track (supported by ballast) takes the form of the letter W depending on the gauge-width and material type. The in-service bending behaviour of plastic composite sleepers with control timber sleepers was studied in the US and it was found that the bending shapes are similar, but it is more prominent in the softer sleepers (plastic) than that of the timber as shown in Figure 3 [43]. This is a similar bending shape of sleepers analysed following the BOEF theory as demonstrated by Qiao et al. [44] when they compared the deflection behaviour of timber and enhanced timber with glass fibre-reinforced plastic (GFRP) wrap. The GFRP-timber sleeper showed less deflection in the rail seat and the ends due to increased stiffness as also shown in Figure 3. This bending behaviour can be mimicked with the five-point bending configuration provided that the load is directly applied to the rail seat area. This not only helps demonstrate the bending behaviour of the sleepers, but also the suitability and integrity of a composite sleeper design as a whole structure can be better understood. This is especially important in the case of composite sleepers with different sectional properties throughout the sleeper length (Figure 1) that is made from different materials along its length and cross section. However, due to the sleeper samples being made of same material throughout their length (commonly available alternative sleeper types) and for the sake of simplicity, the effect of this cross-sectional change is not considered in this research. 

The deflected shape and bending behaviour of sleepers shown in Figure 3 cannot be captured in a single test with three-point or four-point bending configurations. On the other hand, the approach of using a ballast box for full-size testing is both costly and time-consuming due to its big size and heavy weight. Moreover, proper compaction of the ballast is critical to this approach to achieve consistent and comparable test results. Therefore, the five-point bending test could be an alternative but a simple way of mimicking the behaviour of sleepers supported by ballast.

### 3.1. Previous Works on Five-Point Static Bending Test

The five-point bending test is new for railway sleeper applications, and published data on the testing of continuous beams are scarce. Several researchers have successfully applied this testing approach for continuous beams or slabs where the structures are under positive and negative bending moments simultaneously. Kim and Dharan [45] employed this concept for the determination of the interlaminar shear strength of composites. Pouget et al. [46] and Li et al. [47] employed a similar testing method in the study of surfacing systems on orthotropic steel bridges using laboratory-scale samples. Su et al. [48,49] also investigated the behaviour of hollow section aluminium alloy beams under the five-point tests. Mujika et al. [50] modified a three-point bending test into a five-point bending test to do a two-sense bending fatigue test; however, the application was only demonstrated through small-scale laboratory testing. While the five-point bending configuration has been found to be useful by several researchers in evaluating the structural performance of structures, available studies employed a specific testing span, support type and loading type (point load vs. distributed) reflecting the actual application that the test was designed for. A suitable five-point bending test configurations should therefore be determined to best represent the behaviour of sleepers in rail-track. 

### 3.2. Determination of the Appropriate 5-Point Static Test Configuration

**Figure 4:**
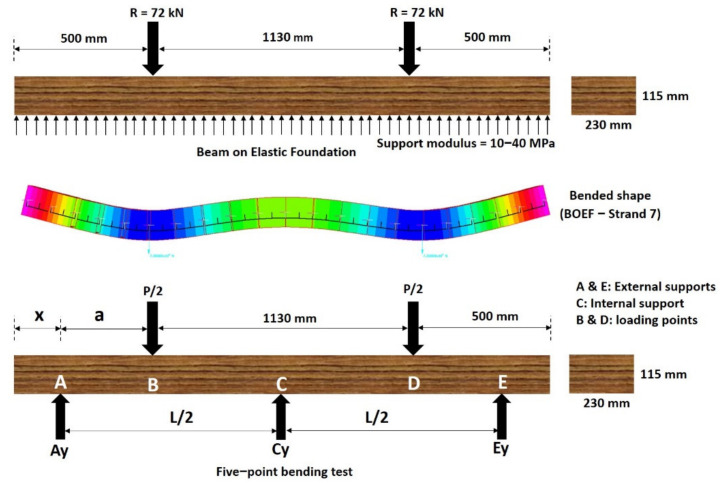
BOEF theory vs. five-point bending test.

**Figure 5:**
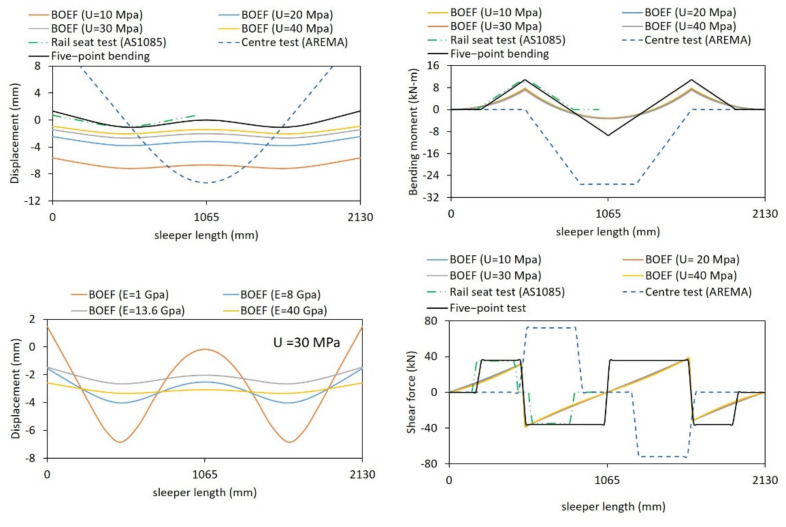
Sleeper behaviour according to BOEF theory, five-point bending, rail seat and centre test.

The five-point bending test, as its name suggests, consists of three supports at the bottom and two loading points on the top (Figure 4). The external or internal span of this bending test changes the intensity of the induced bending moments at the rail seat and the centre of the specimen. Therefore, the distance “*a*” in Figure 4 was carefully determined such that the bending moments at the rail seat and the middle best represent sleepers on ballast. As there is no in-track bending moment data in Australia for timber and its alternatives, BOEF analysis was implemented. A model of each sleeper type has been developed in Strand7 R2.4.6 software from Strand7 Pty Ltd., Sydney, NSW, Australia [51] and is verified with the bending moment equations in Section 4.3.4 of the AS1085.14 [37] with nearly 100% accuracy. This analysis has been based on a typical narrow-gauge Queensland Rail (QR) track configuration in which the distance between the rails is 1130 mm and the sleeper length, L, is 2130 mm [29] with a cross-section of 230 mm (width) by 115 mm (height). Consequently, the distance between the loading points is chosen as 1130 mm so that the positive bending moment is induced at the same location as if the sleeper was in-track. The typical range of the support (ballast) modulus in Australia is between 10 and 40 MPa [40]; however, the BOEF analysis shows that the support modulus value does not affect the bending moment, shear force and deflected shape greatly as shown in Figure 5. On the other hand, changes in the sleeper stiffness greatly affect the deflected shapes of the sleepers. This means that the moment distribution between the rail seat and the sleeper centre is different from a sleeper to another. According to the BOEF analysis, the ratio of positive to negative bending moment for timber sleeper (*E_s_* = 13.6 GPa) is 2.27, with other sleepers being around the same value.

The ratio of positive to negative bending moment for different distances between the external support and the loading point “*a*” is found and compared to the ratios obtained in the BOEF analysis. Compared to the BOEF analysis, the five-point bending test gives lower positive to negative bending moment ratios due to the higher negative bending moment at the centre. However, it was found that a distance of *a* = 300 mm gives the highest bending moment ratios as compared to 350 mm and 400 mm as shown in Table 2. Nevertheless, an “*a*” value shorter than 300 mm would produce high compression at the external supports and the rail seat areas and high shear, which is critical for sleeper technologies with relatively low elastic modulus. Yet, Figure 5 shows that the magnitude of shear force in the five-point bending is similar to the existing AS1085.14 standard, while it is marginally higher than that of the shear force according to BOEF theory. The deflection and bending moment from the five-point bending test setup and BOEF analysis are shown in Figure 5. Figure 5 also compares the behaviour of a timber sleeper under the four-point test method suggested by the Australian standard AS1085.22 [19]. While rail seat bending moments are similar between the five-point and the rail seat bending test, a better match of bending shape to that of the BOEF theory can be captured with the five-point bending test. As the rail seat undergoes the highest bending moment, it is expected that the sleeper failure will occur at the rail seat when loaded ultimately. It has been observed that the in-service, plastic sleepers fail or crack near the rail seats [25]. This also shows the advantage of the five-point bending over the existing four-point bending tests. Besides, the FEA of the centre bending test according to the AREMA standard (also similar to AS 1085) shows that the test induces a much higher bending moment and shear force as compared to the five-point test and the in-service sleepers for the same applied load. A recent study also indicated that none of the standard centre bending tests represents the actual loading conditions of a railway sleeper supported by ballast [27]. This comparative study, therefore, indicates that the five-point bending test could set a foundation for designing bending tests for polymeric railway sleepers. 

Sleeper designs with optimised cross sections and smaller cross-sections in the middle region than at the rail seats may have a lower bending moment capacity at the centre than the rail seat; therefore, these sleepers may first fail in the centre. This behaviour can be captured from the five-point static bending test, but the test setup should properly induce the level of bending moment in the middle region of the sleepers. To overcome this issue, the researchers have used softer materials namely neoprene and EPDM rubber for the middle support to help redistribute the bending moments accordingly. 

## 4. Experimental Verification of the 5-Point Static Bending Test 

### 4.1. Sleeper Properties and Preparation for DIC Measurements

Four sleeper types—hardwood timber, recycled plastic, synthetic composite (SC) and low-profile prestressed concrete sleepers—are tested under the five-point bending setup. The modulus of elasticity of these sleepers are determined using a three-point bending test with a span of 1200 mm on a Universal Testing Machine from Shenzhen SANS Testing Machine Co., Ltd., Guangzhou, China, following the ASTM D790:2017 standard [36], and using the DIC technique for toe compensation (Annex A1 of ASTM D790 [52]). The flexural modulus and sleeper dimensions are listed in Table 3. The timber sleeper is sourced from Queensland, Australia, purchased from Newton Sawmill & Carrying company representing the typical timber sleepers used by the Queensland Rail (QR), while all of the non-timber samples are designed as alternatives to timber sleepers with dimensions suitable for narrow-gauge QR configurations. The hardwood timber sleeper is Grade 1, other species (spotted gum species), and complies with the Queensland Rail’s material supply specification CT.169 [53]. The low-profile concrete sleeper is designed and manufactured by Austrak Pty Ltd., Brisbane, Queensland, Australia following the rational design method [5] with concrete compressive strength of 60 Mpa (28 days) used and contains 20 tendons of low relaxed chevron pattern indentation having a diameter of 5.03 mm each. The synthetic composite (SC) sleeper is a glass fibre-reinforced polyurethane foam type (continuously reinforced in the longitudinal direction) and is supplied by AGICO Group Company, Anyang, Henan, China. The plastic sleeper is manufactured and supplied by Replas plastic recycling company in Melbourne, Victoria, Australia, and it is made of post-consumer recycled plastics with fillers.

The DIC method is a versatile and effective non-contact full-field technique of measurement that has been employed in various polymer composite research [54,55,56,57]. Sui et al. (2018) [58] indicated that DIC technology is an accurate way of measuring full deflection profiles of beams under flexural bending. Xian-rong [59] highlighted that using a single camera correlation technique for deflection measurement can result in an accuracy of 0.1 mm. In addition, Sladek et al. [60] found a difference of only 0.174 mm (minimum 0.010 mm with a mean difference of 0.063 mm) in the deflection measurements of an optical laser and a single camera DIC technique. Accordingly, the single-camera DIC technique was implemented, as this seems a suitable method to capture the full deflection profile of the sleepers under the five-point static bending test. 

**Figure 6:**
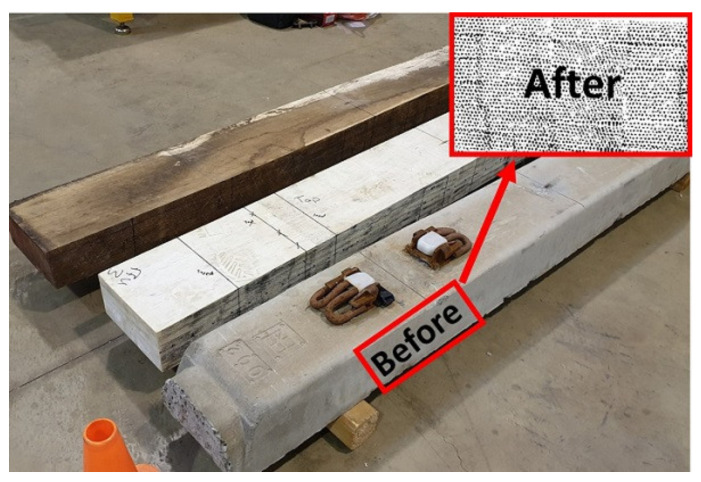
Sleeper samples showing applied speckle pattern.

The DIC method in this research was used to measure the displacement at the supports and the deformed shape of the sleepers along its entire length for comparison with the results from the BOEF analysis. Before testing, all sleepers were painted white and randomly speckled with black ink on the observation side as shown in Figure 6 for the DIC measurement. The random speckle pattern helps with pixel tracking (displacement measurement) as the system uses the 256 levels of greyscale for digitisation of the black and white image considering the light intensity. By default, the DIC system measures displacement with respect to image pixel location and hence it requires calibration or a referencing system to real units. This was achieved by drawing 100 mm squares on the plane of the measurement (observation face of the sleepers) and then calibrating it in Video Gauge software [61]. 

### 4.2. Non-Destructive Five-Point Static Bending Tests

**Figure 7:**
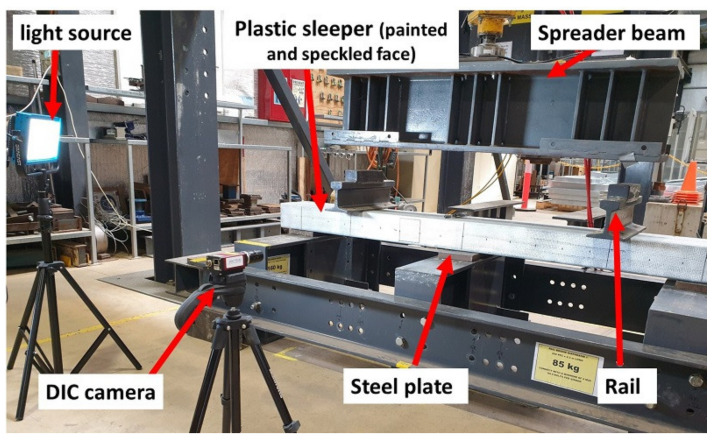
Actual test setup of the five-point bending test, showing the plastic sleeper.

As shown in Table 2, the default all-steel support five-point bending test with the shear span of 300 mm produces a high bending moment at the centre as compared to that of the results from the BOEF analysis. The use of resilient pads at the middle support introduces support settlement to flatten the bent shape of the sleepers, thus reducing the centre bending moment. As available timber replacement sleepers have a wide range of elastic modulus, it was expected that the sleepers would have different responses to the softer middle support in terms of moment reduction. Accordingly, two different elastic supports, namely, neoprene and EPDM rubber with a thickness of 25 mm and a width of 150 mm, were considered in addition to steel plates. The external supports are of a steel type of 25 mm thickness and 150 mm width in all cases. The neoprene rubber is a shore A hardness 90 type specified in the bending moment tests for prestressed concrete and alternative sleeper materials [19,37]. The EPDM rubber is a commercially available sealing rubber in Australia with a Shore A hardness of 45 to 60 as reported in the literature [62,63,64,65]. According to Ferdous et al. [29], the approximate rail seat load for a timber track based on a 20-ton axle wheel load is 72 kN. Accordingly, a total load of 144 kN was applied to the samples through a spreader beam resting on two rail sections of 1130 mm apart mimicking the narrow-gauge track in Queensland as shown in Figure 7. 

**Figure 8:**
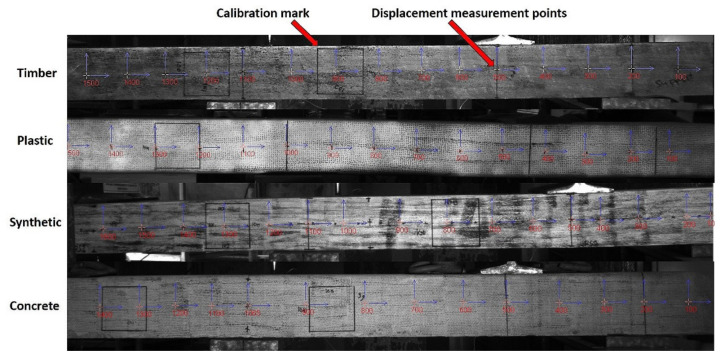
Digital Image Correlation (DIC) images of the sleepers showing displacement points (neoprene support at centre).

The deflected shape of the sleepers along their length was captured using the DIC camera. A screenshot of the DIC image for each sleeper type is provided in Figure 8. An LVDT instrument was also used to measure the rail seat displacements for validation of the measured displacement using the DIC. From the measured settlement at the middle support (using the DIC), the positive and negative bending moments can be calculated. The ratios of the positive-to-negative bending moments, compared to the BOEF, were then calculated and used as a basis to evaluate the most suitable test configuration that best represents the flexural behaviour of railway sleepers supported by ballast. 

## 5. Results and Discussion

This section presents the experimental results of the full-scale five-point static bending test of different timber alternative sleeper technologies. The load and displacement relationship curves of the rail seat and the centre of the sleepers are presented, highlighting the differences in the settlement of the middle support when resilient pads were used. The deflection profiles of the sleepers along its length measured from the experimental test, and the results of the BOEF analysis are presented. Moreover, the effect of the materials used for the middle support and the modulus of elasticity of the sleepers were analysed and discussed.

### 5.1. Effect of Materials at the Middle Support

**Figure 9:**
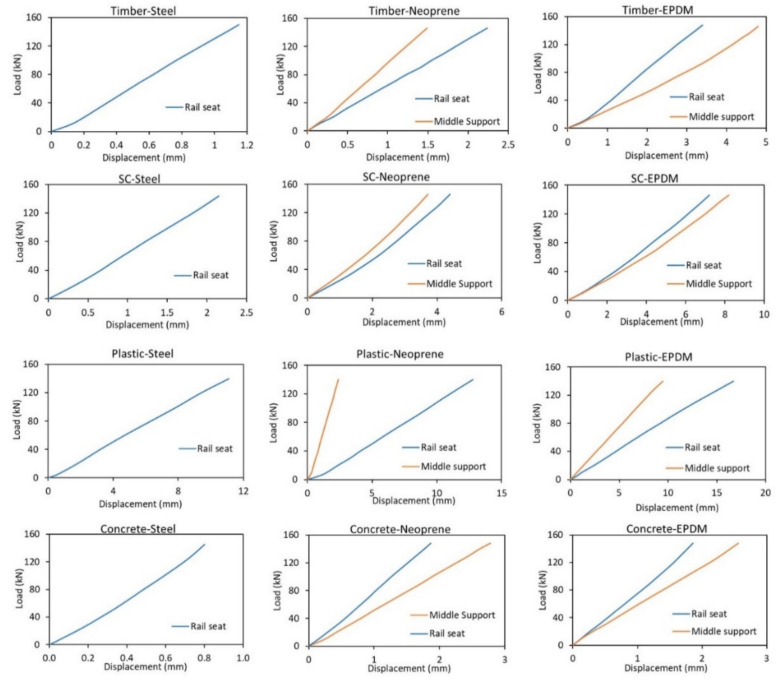
Load–displacement graphs measured at rail seat and centre.

**Figure 10:**
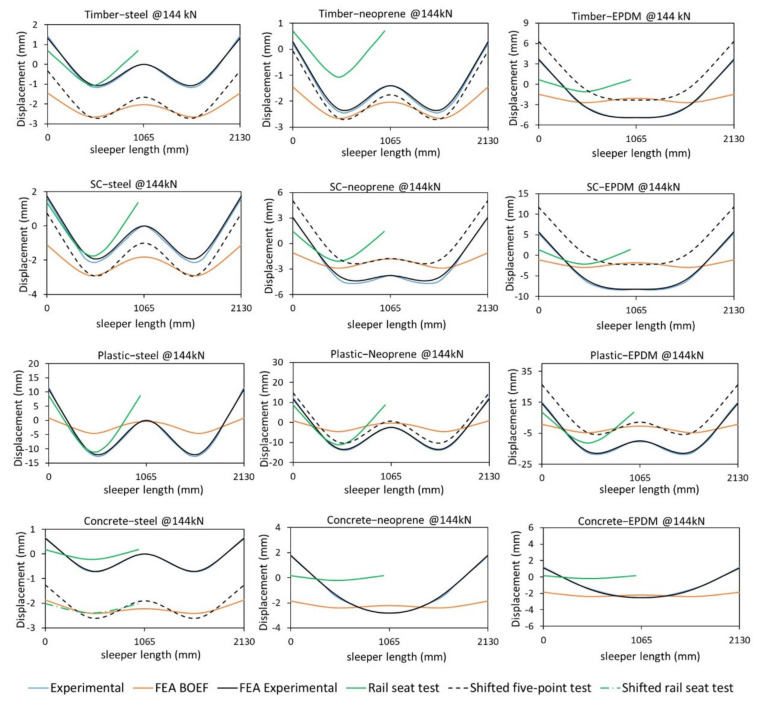
Full-length deflection shape of the sleepers for different support types.

Figure 9 shows the load–displacement behaviour of the sleepers at the rail seat and at the centre, measured using the DIC camera. From the level of load applied, no failure was observed for all the sleepers. The displacement readings from the LVDT at the rail seats are exactly similar to that of the measured displacement from DIC for all sleeper samples. Figure 10 compares the deflected shape according to the five-point static bending test with different materials at the middle support, the BOEF theory and the rail seat test according to AS1085.22 [19] at a service load of 144 kN. 

Compared to all-steel supports, the rail seat deflections increased noticeably for all sleeper types when neoprene and EPDM rubber were used at the middle support. The middle support settlement using EPDM is higher than that of the neoprene for all sleeper types with zero settlement using steel at the middle support. Although the use of a softer material at the middle support will introduce more settlement in the sleeper’s centre, it will flatten the deflected shape at the centre. This higher settlement value however does not necessarily mean that this support type will reduce the bending moment at the centre. This middle support settlement must be compared to the rail seat deflection and the bending moment envelope from the results of the BOEF analysis. If the middle support settlement is higher than that of the rail seat, this means that the test configuration failed to induce a negative bending moment in the centre as in the case of the concrete sleeper, or the negative bending moment is significantly low as in the case of timber and SC sleepers. This behaviour can be explained further by comparing the deflected shape of the sleepers along their entire length and tested with different support types. 

Figure 10 indicates that for most of the tests, the W-shaped bending behaviour was captured. However, differences can be seen between the theoretical (BOEF) and the experimental results. This difference is because, in reality, sleepers are supported by a continuous ballast which results in a gradual bending shape (more subtle). In the bending test, however, the bending profile is more noticeable due to the smaller support areas (three points only). Despite these differences, the success of this test is measured by comparing the intensity of the bending moments at both the rail seat and the centre of the sleepers together with the bending shape profiles. The following paragraphs discuss the bending shape similarity, while the next section discusses the bending moment behaviours. 

From Figure 10, it is obvious that the EPDM rubber support is not a suitable material for the middle support for timber, SC and concrete sleepers, as it failed to reproduce a bending moment envelope similar to that of the BOEF as well as very low value of negative bending moment (hogging) at the centre. This is supported by the findings of Carrasco et al. (2012) [66] that sleepers of similar bending modulus would show a clear W-shape bending behaviour, meaning a hogging moment is expected for these sleepers under similar load. This behaviour is caused by the big differences between the stiffness of the resilient pad and the sleepers. Due to the soft EPDM pad, this material kept deflecting (compressing) throughout the test and could not resist the stiffer sleeper material from deflecting at midspan, thus not inducing a negative bending moment. The difference in the FE analytical and the experimental results of the concrete sleepers can be explained similarly for EPDM and neoprene support types. The stiffness of the concrete is so high that the material (EPD or neoprene) of the middle support could not resist the deflection at the centre of the concrete, thus the bending shape is U-shaped. Another reason for this difference could be because of the continuous elastic support in the case of the FEA, whereas in the experimental case the sleeper was supported by two stiff steel supports (external) and one soft internal support. The obtained W-shape deflection of concrete sleepers for steel-steel support also confirms this claim where all the supports have the same stiffness (rigid steel). Out of the tree configurations, the steel-steel support is deemed most suitable for sleepers with very high stiffness (*E_s_* = 38.1 GPa) as the deflection behaviour of the concrete sleeper with all-steel supports shows the best match to that of the BOEF theory (compared to other support types). This can be supported by the findings in [24,34], where the authors indicated that sleepers of very high bending modulus still show a negative bending moment at the centre but it is considerably lower than that of the rail seat. This behaviour could only be captured using the all-steel support as the centre part of the concrete sleepers was considerably flatter than the rail seat section. However, the bending behaviour according to the existing rail seat test shows a better match as compared to the five-point bending. Note that for a higher level of loading (say ultimate load), this may change as the centre steel support does not deflect but the rail seat deflection would increase leading to a more similar behaviour to that of the AS1085.14 standard and the BOEF theory. 

There is a slight variation in the bending shape of the timber-steel and timber-neoprene support with the latter being more suitable due to the increased bending at the rail seat and thus increased bending moment. This is due to the settlement of the middle support (EPDM) which resulted in a slightly flatter bent sleeper shape in the centre. It can also be said that neoprene is a more suitable support for SC sleepers due to a flatter centre bent shape which results in a much lower bending moment at the centre than that of the rail seat. On the other hand, the deflected shape of the plastic sleeper shows that the EPDM rubber is the most suitable and closest match to that of the results from BOEF. This means that the EPDM pad, despite its very low stiffness, can resist the bending effect of the plastic sleeper because of its compatible low stiffness (*E_s_* = 1.0 GPa). This high deflection (and thus clear W-shape bending) of plastic sleepers is also noticed from field measurements which require less load to induce the same amount of deflection as timber sleepers [43]. In conclusion, it was found that with the existing span configurations, the most suitable support type for timber and SC sleepers is steel-neoprene, for plastic sleepers is steel-EPDM and for concrete sleepers is steel-steel type. 

### 5.2. Effect of Sleeper Stiffness

**Figure 11:**
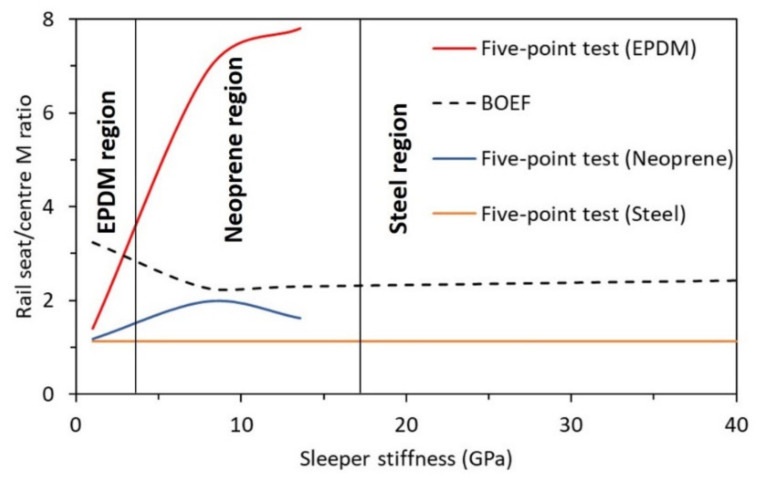
Relationship of positive/negative moment ratio with sleeper stiffness.

Figure 11 illustrates the change in the positive to negative bending moment ratios with the increase in the sleeper stiffness tested with different support types. Equations (1)–(4) were used to calculate the positive to negative bending moment ratios of sleepers supported by all-steel, steel-EPDM and steel-neoprene. These values were then plotted against the corresponding modulus of elasticity of the sleepers to obtain the curves in Figure 11. The plot of the BOEF is based on the average values for a typical ballast modulus range in Australia (10–40 MPa) [40]. Figure 11 helps visualise how the change in the sleeper stiffness affects the bending moment ratios at the rail seat and sleeper centre for different support types. This information makes it easier to compare the closeness of the five-point bending test to that of BOEF for each support type and to evaluate the suitability of the materials used at the middle support for railway sleeper with a different modulus of elasticity (see Table 4). 

In Figure 11, three regions based on middle support types and sleeper’s modulus of elasticity can be derived. It can be seen that the EPDM rubber support is most suitable for sleeper with *E_s_* ranging from 1 to 4 GPa, the neoprene rubber is most suitable for *E_s_* ranging from 4 to 17 GPa and steel support is most suitable for sleepers with *E_s_* of 17 GPa and higher. However, note that these results are for sleepers with a moment of inertia within those reported in Table 3. The values of the bending moment at the rail seat and the centre of the sleepers tested with different middle supports are tabulated in Table 4. While the rail seat to centre bending moment ratio in the second region (4 to 17 GPa) is closest to the ratio-based BOEF, it is expected that EPDM rubber support for sleeper with *E_s_* of ~2.5 GPa would show the closest behaviour to that of the results from the BOEF. When it comes to standardising the five-point bending test, neoprene support could satisfy the requirements of most polymeric railway sleepers. This is because most of the alternative polymer sleepers have a modulus of elasticity within the second bending modulus range illustrated in Figure 11.

Table 4 emphasises the most suitable middle support for a specific sleeper type according to bending moment behaviours. The decision process (column 7 of Table 4) was based on the comparison of the moment ratios of the test and the BOEF theory. For example, the positive-to-negative bending moment ratio of the synthetic sleeper is 1.99 when EPDM support is used, while this ratio is 6.92 when neoprene is used as the middle support. Considering that this ratio for the synthetic sleeper is 2.23 according to the BOEF, it is evident that the EPDM best replicated this behaviour than the neoprene and the steel (steel ratio = 1.14) supports. 

## 6. Analytical Solution of Five-Point Bending and FEA Verification

Table 4 contains the bending moment calculation at the rail seat and centre of the sleepers. As the five-point bending setup is statically indeterminate, two moment equations based on indeterminate beam analysis theories were derived and used for these calculations. This section presents the analytical solution of the five-point bending test set-up with and without middle support settlement using beam theory. The verification of these equations is also carried out using finite element analysis in Ansys Workbench software.

The five-point bending test setup can be represented simply as a supported beam with extra roller support at the centre of the beam or as a continuous beam of two spans as shown in Figure 4. The middle support introduces a new vertical reaction and a negative bending moment in the centre of the beam. 

Using equilibrium theory, the general moment equations at the rail seat (*M_B_*) and the centre (*M_C_*) can be expressed as

*M_B_* = *A_y_ AB* (1)

*M_C_* = *A_y_ AB* − (*P*/2 − *A_y_*) *BC* (2)

where *AB* is the distance between points *A* and *B*, and *BC* is the distance between points *B* and *C*. *P* is the total load applied to the sleeper. The unknown reaction at support *A* (*A_y_*) in Equations (1) and (2) cannot simply be calculated from the equilibrium theory due to the continuity of the beam (extra support at the centre and thus the reaction *C_y_*). When *C_y_* is calculated, the external reactions (*A_y_* and *E_y_*) can easily be calculated from symmetry (*A_y_* = *E_y_*). Therefore, an indeterminate beam analysis method can be applied to calculate the middle reaction *C_y_*.

There are several methods of indeterminate beam analysis such as force method, displacement method (slope deflection and moment distribution) and direct stiffness method [67]. The force method, which is also called the consistent deformation method, is considered in the analysis of the indeterminate beam due to its direct relevance and applicability to the 5-point test configuration for sleepers. Similarly, the method of superposition is adopted due to its simplicity. To generate the compatibility equations in the force method, the sleeper is represented with two separate determinate beams as also implemented by [68]. The first beam, which is also called the basis beam, represents the whole sleeper (setup) without the middle support. The second beam, which is called the redundant beam, is a simply supported beam with an upward vertical force (*C_y_*) acting at the middle of the beam (the two rail seat loads are removed), accounting for the middle support effect which was removed in the first (basis) beam. The compatibility equation can now be obtained through establishing the continuity of deformation between the basis beam and the redundant beam for the middle support. When the middle support is removed, the deflection at mid-span (point *C*) can be written as *δC*_1_ = − *Pa* (3*L*^2^ − 4*a*^2^)/(48 *E_s_ I).* In this relation, *E_s_* is the modulus of elasticity of the sleeper in the longitudinal direction, *I* is the second moment of inertia of the sleeper and *L* is the distance between the external supports A and E. For the redundant member, the mid-span deflection due to the upward force (*C_y_*) can be written as *δC*_2_ *= (C_y_ L*^3^)/(48 *E_s_ I).* Writing the compatibility equation for the middle support, i.e., *δC*_1_ + *δC*_2_ = 0, then

−*Pa* (3*L*^2^ − 4*a*^2^)/(48 *Es I*) + *C_y_ L*^3/^(48 *Es I*) = 0

∴ *C_y_* = *Pa* (3*L*^2^ − *4a*^2^)/*L*^3^ (3)

In Equation (3), *a* is the distance between the rail seat and the external support. 

For the five-point static bending tests with elastic support at the centre, there is a settlement at the middle support. Therefore, the summation of the displacements in the compatibility equation is not equal to zero and can be written as

−*Pa* (3*L*^2^ − 4*a*^2^)/(48 *Es I*) + *C_y_ L*^3/^(48 *Es I*) = −Δ*C_R_*

where Δ*C_R_* is the relative displacement of the middle support with reference to the external supports. Equation (3) then becomes

*C_y_* = (*Pa* (3*L*^2^ − 4*a*^2^) − 48 *Es I* Δ*C_R_*)/*L*^3^ (4)

**Figure 12:**
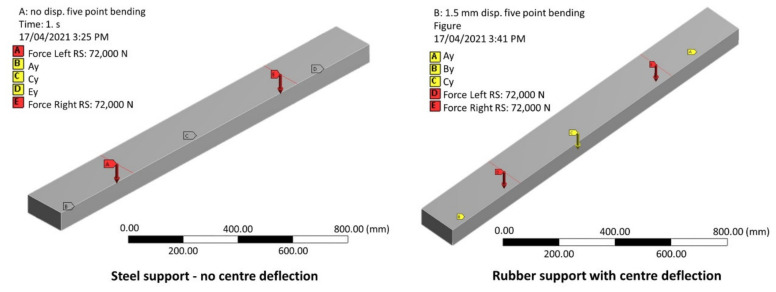
Validation of the analytical solution with finite element (FE) analysis.

To validate the analytical solution, a three-dimensional FEA based on a typical QR timber sleeper has been conducted (Figure 12) using Ansys Workbench 19.2 software, from Ansys, Inc. Canonsburg, Pennsylvania, US. The sleeper was modelled using Solid186 homogenous structural solid element. The sleeper has an elastic modulus of 13.6 GPa and a cross-section of 230 mm by 115 mm. A bending strength of 55 MPa, a tensile strength of 34 MPa (parallel to grain) and a compression strength of 42 MPa (parallel to grain) were considered based on the AS 1720.1 [69]. The total load applied is 144 kN (72 kN per rail seat). The external supports were restrained for movement in all directions; however, rotation along the longitudinal axis was set to free to allow for the bending effect. To validate Equation (2), a relative displacement of 1.5 mm (Δ*C_R_*) was applied to the middle support. The support reactions and the bending moments according to the analytical and the results of the numerical solutions are compared in Table 5.

The numerical solution shows a very good agreement with the analytical solution. The small variations could be due to the compression of the sleeper at the supports and the loading points for the numerical analysis due to the three-dimensional shape. The experimental results were also compared to that of the results of the FE analysis to ensure the bending moments calculated according to the analytical equations represent the experimental results. This was achieved by monitoring the strain redistribution at the rail seat and the centre due to the middle support settlement using the DIC technique. The measurement was obtained on the front face of the sleepers, i.e., bottom rail seat and top centre, where maximum bending moments expected to occur. The strain measurement was justified because the sleeper materials were stressed within their elastic region, as the load-displacement graphs (Figure 9) also demonstrates. This means that the ratio of rail seat to centre strain represents the stress ratio and thus the bending moments for a specific load (144 kN in this case). It was found that the rail seat to centre strain ratio is 1.2 for all steel supports, while it increased to 1.5 when neoprene support used at the centre. Although there is a difference of 0.1 when compared to the bending moment ratios in Table 5, this difference is justifiable as the strain was measured on the front face of the sample, not on the bottom (for rail seat) and top (for centre) faces where maximum strain occurs. Notwithstanding, it is clear that when neoprene support was used, the rail seat strain and thus the bending moment increased, as also shown in Figure 10, where the bending shape at the rail sear is much sharper when neoprene support is used. 

Therefore, Equations (1)–(4) can be used to analyse the five-point static bending tests and predict the bending moment distribution along the sleeper for different loading levels and support types with measured relative middle support settlement. This information is useful in the evaluation of the performance of the sleepers as well as in future development. For example, a middle-support settlement of 1.5 mm will result in a middle-support reaction reduction from 71.91 kN to 66.40 kN calculated using Equation (4) (for timber sleeper, i.e., *E_s_* = 13.6 Gpa). Using this value, the magnitude of the external support (*A_y_*) can be calculated as well as the magnitude of the centre bending moment (Equation (2)). A summary of the magnitude of the centre bending moment for timber sleeper under an applied rail seat load of 72 kN is provided in Table 5. Note that the compression of the sleepers at the rail seat and the supports is not considered in the analysis. Finally, any changes regarding the test span because of different gauge-width can be incorporated into the equation simply by changing the values of “*a*” and “*L*”. 

## 7. Conclusions

This paper presents a new test method for evaluating the flexural behaviour of timber-alternative sleeper technologies. The effectiveness of the five-point static bending test was evaluated by non-destructive testing of four sleeper types having different modulus of elasticity: timber (*E_s_* = 13.6 GPa), recycled plastic (*E_s_* = 1.0 GPa), synthetic composites (*E_s_* = 8.1 GPa) and low-profile prestressed concrete (*E_s_* = 38.0 GPa) sleepers. Moreover, three materials were considered in the middle support—steel, neoprene and EPDM rubber pads, with the end supports using steel in the verification of the five-point static bending test. From the results of this work, the following conclusions can be made:

- The five-point static bending test is a simple test method to simulate the sleeper behaviour supported by ballast and subject to simultaneous positive and negative bending moments. The closeness of this testing method to that of the in-situ situation is limited to the sleeper behaviour according to BOEF theory and the shear span of 300 mm to prevent high shear stress beyond that of the AS1085.14 standard. The deflected profiles from the five-point static bending test are very similar to that of the deformation behaviour from analysis using the beam on elastic foundation except for the concrete sleepers.
- The bending modulus of the sleeper is a more influential parameter than the support modulus (ballast) when determining the bending moment, shear force and deflected shape of the sleepers. The ratio of bending moment at the rail seat (sagging) to the centre bending moment (hogging) increases with the increase in the modulus of elasticity of the sleepers. The sagging to hogging moment ratio of recycled plastic sleeper (*E_s_* = 1.0 GPa) increases due to higher bending at the rail seat due to its significantly low elastic modulus.
- The hardness or elasticity of the middle support in a 5-point bending test has a significant influence in inducing appropriately the magnitude of the positive and negative bending moments experienced by railway sleepers. Neoprene rubber is found suitable for timber and FFU sleepers, EPDM rubber seemed suitable for plastic sleepers and steel support for low-profile prestressed concrete sleepers. This indicates the type of middle support is very much dependant on the elastic modulus of the sleeper materials, i.e., the higher the elastic modulus of the sleeper, the stronger the middle support material is required. Neoprene support is however suggested to standardise the five-point bending for polymeric-based railway sleepers.
- The modulus of railway sleepers directly affects the bending moment distribution between the rail seat and centre of the sleepers. The positive-to-negative bending moment increases as the sleeper stiffness increases for neoprene and EPDM support. The high elasticity of the low-profile prestressed concrete sleeper requires a steel pad to induce a negative bending moment at the middle of the sleeper. This was however limited to the loading intensity and type (static) applied in this investigation.
- The developed theoretical equation based on the force method analysis of indeterminate beam and considering the settlement of the middle support and modulus of elasticity of the sleepers can calculate directly the reactions at supports and bending moments along the length of the sleeper. The verification with FEA analysis for timber sleeper showed that the analytical solution can accurately predict the magnitude of the bending moments at the rail seat and centre of the sleeper under 5-point static bending tests.

The above results showed that the 5-point static bending test is a simple and reliable testing method to evaluate the bending behaviour of timber alternative sleeper technologies. Further research is however required to evaluate the effectiveness of this testing method for ultimate and cyclic loading conditions, especially to confirm the failure location and behaviour of different polymer-based sleeper technologies. Moreover, the effectiveness of this new test method in evaluating the bending performance of other alternative sleeper technologies beyond those considered in this study should be conducted. For example, sleepers with different material properties (thus different *E_s_*) or different sectional properties along the length of the sleepers. Once this is achieved, this new test method can lead to the development of a unified test standard for current and emerging railway sleeper technologies.

## Figures and Tables

**Table 1:** Comparison of different alternative sleeper standards.

	Origin Country	Sleeper/Material Type Co	E_s_ (GPa)	Full-Size Bending Test	Type of Resilient Support
AREMA- chapter 30-5	USA	ECP and EWP, but mostly deal with HDPE polymer-based composite products.	1.17	Rail seat positive, rail seat negative and centre negative.	140 mm × width of sleeper × 25 mm thick (50 Shore A hardness)
JIS E 1203:2007	Japan	Fibre-reinforced foamed urethane.	6.0	No full-size testing	-
ISO 12856	International	Plastic and reinforced plastic	1.17–6.0	Rail seat positive, centre positive and centre negative.	140 mm × width of sleeper × 15 mm thick (static bedding modulus: 1 < C < 4 N/mm^3^)
AS1085.22	Australia	Not specified	-	Rail seat positive, rail seat negative, centre positive and centre negative.	Neoprene Shore A hardness 90. Top: 25 mm width × 12 mm thick × width of sleeper Bottom: 50 mm width × 25 mm thick × width of sleeper

**Table 2:** Positive and negative bending moments of different “*a*” values.

Distance ‘*a*’	Bending Moment (kN-m)		
	Positive	Negative	Ratio
BOEF (timber)	7.3	3.22	2.27
400 mm	11.91	11.92	0.999
350 mm	11.44	10.76	1.063
300 mm	10.81	9.5	1.137

**Table 3:** Properties of the sleeper samples.

Sleeper Type	Cross-Sectional Area (mm^2^)	Length (mm)	Second Moment of Inertia (mm^4^)	Es (GPa)
Timber	26,450	2130	29,150,104	13.6
Recycled plastic	28,125	2050	36,621,094	1.0
Synthetic composite	25,760	2120	28,389,667	8.1
Prestressed concrete	31,168	2130	48,699,500	38.0

**Table 4:** Bending moment values for different support types (B.M: Bending moment; RS: Rail seat).

Sleeper Type	Support Type	Middle Support Settlement (ΔCR) mm	B.M @ RS (kN-m)	B.M @ Centre (kN-m)	RS/Centre B.M Ratio	Remarks/Most Suitable Support
Timber	Steel	0.00	10.81	−9.50	1.14	Low
	EPDM	4.89	13.51	−1.73	7.81	High
	Neoprene	1.50	11.64	−7.12	1.64	✓
	BOEF	-	7.3	−3.22	2.27	Target
Plastic	Steel	0.00	10.81	−9.50	1.14	Low
	EPDM	9.80	11.32	−8.10	1.40	✓
	Neoprene	2.40	10.94	−9.15	1.20	Low
	BOEF	-	4.94	−1.6	3.09	Target
Synthetic	Steel	0.00	10.81	−9.5	1.14	Low
	EPDM	8.20	13.43	−1.94	6.92	High
	Neoprene	3.75	12.01	−6.04	1.99	✓
	BOEF	-	6.94	−3.11	2.23	Target
Concrete	Steel	0.00	10.81	−9.5	1.14	✓
	EPDM	2.80	18.40	12.36	-	‘No negative moment’
	Neoprene	2.55	17.72	10.42	-	
	BOEF	-	8.00	−3.41	2.34	Target

**Table 5:** Comparison of analytical and numerical solutions.

Middle Support Condition	Type of Analysis	Ay and Ey (kN)	Cy (kN)	Moment at Rail Seats, RS (kN-m)	Moment at Centre, C (kN-m)	RS/C Ratio
No settlement	Analytical	36.05	71.91	10.81	−9.50	−1.138
	FEA	36.07	71.85	10.82	−9.47	−1.142
1.5 mm settlement	Analytical	38.80	66.40	11.64	−7.12	−1.635
	FEA	38.70	66.59	11.61	−7.20	−1.613

## References

[1] Zakeri J.-A., Rezvani F.H. (2012). Failures of railway concrete sleepers during service life. Int. J. Constr. Eng. Manag..

[2] Ferdous W., Manalo A., Van Erp G., Aravinthan T., Kaewunruen S., Remennikov A. (2015). Composite railway sleepers–Recent developments, challenges and future prospects. Compos. Struct..

[3] Nosker T., Renfree R., Lynch J., Lutz M., Gillespie B., Lampo R., Van Ness K.E. (1998). A performance-based approach to the development of a recycled plastic/composite crosstie. Proceedings of the Technical Papers of The Annual Technical Conference-Society of Plastics Engineers Incorporated.

[4] Ju S., Yoon J., Sung D., Pyo S. (2020). Mechanical Properties of Coal Ash Particle-Reinforced Recycled Plastic-Based Composites for Sustainable Railway Sleepers. Polymers.

[5] Nairn J., Stevens N. (2010). Rational design method for prestressed concrete sleepers. CORE 2010: Rail, Rejuvenation and Renaissance.

[6] Ahn S., Kwon S., Hwang Y.-T., Koh H.-I., Kim H.-S., Park J. (2019). Complex structured polymer concrete sleeper for rolling noise reduction of high-speed train system. Compos. Struct..

[7] Brown J. (1979). Design refinements make the steel sleeper viable. Railw. Gaz. Int..

[8] Manalo A., Aravinthan T., Karunasena W., Ticoalu A. (2010). A review of alternative materials for replacing existing timber sleepers. Compos. Struct..

[9] Shokrieh M.M., Rahmat M. (2006). On the reinforcement of concrete sleepers by composite materials. Compos. Struct..

[10] Lampo R., Nosker T., Sullivan H. Development, testing, and applications of recycled-plastic composite cross ties. Proceedings of the DoD Transportation 2000 Workshop.

[11] Jeon E.-B., Ahn S., Lee I.-G., Koh H.-I., Park J., Kim H.-S. (2015). Investigation of mechanical/dynamic properties of carbon fiber reinforced polymer concrete for low noise railway slab. Compos. Struct..

[12] (2020). Composites in the Passenger Rail Market Report: Trends, Forecast and Competitive Analysis, Market Share Report.

[13] (2016). Expression of Interest for Alternative Composite Sleeper Product, Tender Number: QRP15150A.

[14] Silva É.A., Pokropski D., You R., Kaewunruen S. (2017). Comparison of structural design methods for railway composites and plastic sleepers and bearers. Aust. J. Struct. Eng..

[15] Qiu C., Tew G., Trevaskis R. (2018). The application of composite sleepers contributing to the sustainability of railway: Material performance and standard development. Proceedings of the CORE 2018: Conference on Railway Excellence.

[16] AREMA (2014). Chapter 30—Ties.

[17] Japanese Industrial Standard (2007). JIS E 1203:2007, Synthetic Sleepers—Made from Fibre Reinforced Foamed Urethane.

[18] International Organization for Standardization (2014). ISO 12856-1: Plastics—Plastic Railway Sleepers for Railway Applications (Railroad ties).

[19] Standard Australia (2020). AS 1085.22: 2019, Railway Track Materials: Alternative Material Sleepers, RISSB.

[20] Zakeri J.A., Sadeghi J. (2007). Field investigation on load distribution and deflections of railway track sleepers. J. Mech. Sci. Technol..

[21] Kaewunruen S., Remennikov A.M. (2008). Effect of a large asymmetrical wheel burden on flexural response and failure of railway concrete sleepers in track systems. Eng. Fail. Anal..

[22] Edwards J.R., Gao Z., Wolf H.E., Dersch M.S., Qian Y. (2017). Quantification of concrete railway sleeper bending moments using surface strain gauges. Measurement.

[23] Van Dyk B. (2015). Characterization of the Loading Environment for Shared-Use Railway Superstructure in North America.

[24] Wolf H.E., Edwards J.R., Dersch M.S., Qian Y., Lange D.A. Field measurement of bending moments in prestressed concrete monoblock sleepers. Proceedings of the 11th World Congress Railway Research.

[25] McHenry M., Gao Y., Billargeon J.P. (2018). Implementing Improved Composite Tie Design and Testing Guidelines. Proceedings of the American Railway Engineering and Maintenance-of-Way Association Annual Conference.

[26] Bolin C.A., Smith S.T. (2013). Life cycle assessment of creosote-treated wooden railroad crossties in the US with comparisons to concrete and plastic composite railroad crossties. J. Transp. Technol..

[27] Kaewunruen S., Ngamkhanong C., Sengsri P., Ishida M. (2020). On Hogging Bending Test Specifications of Railway Composite Sleepers and Bearers. Front. Built Environ..

[28] Aravinthan T., Manalo A. Field applications and case studies of FRP in civil infrastructure: The Australian experience. Proceedings of the 6th International Conference on FRP Composites in Civil Engineering (CICE 2012).

[29] Ferdous W., Manalo A., Van Erp G., Aravinthan T., Ghabraie K. (2018). Evaluation of an innovative composite railway sleeper for a narrow-gauge track under static load. J. Compos. Constr..

[30] Van Erp G., Mckay M. (2013). Recent Australian developments in fibre composite railway sleepers. Electron. J. Struct. Eng..

[31] KLP® Hybrid Polymer Sleepers. https://www.hirdrail.com/klp-polymer-sleepers.html.

[32] Nose G.L., Trevizan D.W. (2020). Railway Sleeper and Railway-Sleeper Manufacturing Method. U.S. Patent.

[33] Zakeri J.A., Fattahi M., Ghanimoghadam M.M. (2015). Influence of unsupported and partially supported sleepers on dynamic responses of train–track interaction. J. Mech. Sci. Technol..

[34] Shokrieh M.M., Rahmat M. (2007). Effects of Young’s modulus on response of railway sleeper. Appl. Math. Model..

[35] Ferro E., Harkness J., Le Pen L. (2020). The influence of sleeper material characteristics on railway track behaviour: Concrete vs. composite sleeper. Transp. Geotech..

[36] Abadi T., Pen L.L., Zervos A., Powrie W. (2019). Effect of Sleeper Interventions on Railway Track Performance. J. Geotech. Geoenviron. Eng..

[37] Standards Australia (2012). AS 1085.14-2012: Railway Track Material. Prestressed Concrete Sleepers.

[38] International Organization for Standardization (2018). ISO 12856-2, Railway Applications-Polymeric Composite Sleepers, Bearers and Transoms.

[39] Janeliukstis R., Clark A., Papaelias M., Kaewunruen S. (2019). Flexural cracking-induced acoustic emission peak frequency shift in railway prestressed concrete sleepers. Eng. Struct..

[40] Jeffs T., Tew G. (1991). A Review of Track Design Procedures: Sleepers and Ballast.

[41] Hetenyi M. (1967). Beams on Elastic Foundation.

[42] Sadeghi J., Barati P. (2010). Evaluation of conventional methods in Analysis and Design of Railway Track System. Int. J. Civ. Eng..

[43] U.S. Department of Transportation, Federal Railroad Administration (2011). Performance of Plastic Composite Ties in Revenue Service.

[44] Qiao P., Davalos J.F., Zipfel M.G. (1998). Modeling and optimal design of composite-reinforced wood railroad crosstie. Compos. Struct..

[45] Kim W., Dharan C. (1995). Analysis of five-point bending for determination of the interlaminar shear strength of unidirectional composite materials. Compos. Struct..

[46] Pouget S., Sauzéat C., Di Benedetto H., Olard F. (2010). Numerical simulation of the five-point bending test designed to study bituminous wearing courses on orthotropic steel bridge. Mater. Struct..

[47] Li J., Liu X., Scarpas A., Tzimiris G., Kasbergen C., Hofman R., Voskuilen J. (2013). Analysis of five-point bending test for multilayer surfacing system on orthotropic steel bridges. Transp. Res. Rec..

[48] Su M.-N., Young B., Gardner L. (2015). Continuous beams of aluminum alloy tubular cross sections. I: Tests and FE model validation. J. Struct. Eng..

[49] Su M.-N., Young B., Gardner L. (2015). Continuous beams of aluminum alloy tubular cross sections. II: Parametric study and design. J. Struct. Eng..

[50] Mujika F., Asensio M., Vargas G., Boyano A., De Gracia J. (2015). Analysis of a reversible five-point bending configuration based on a novel two-sense support. Polym. Test..

[51] Strand7 Software, Strand7 Release 2.4.6. https://www.strand7.com/WhatsNew/release240.htm.

[52] (2017). ASTM D790-17—Standard Test Methods for Flexural Properties of Unreinforced and Reinforced Plastics and Electrical Insulating Materials.

[53] Queensland Rail (2018). Material Supply Specification TRACK-CT.169. Civil—Sleepers—115 mm and 150 mm Thickness.

[54] Yu L., Pan B. (2018). Experimental study of tensile properties and deformation evolutions of 2D and 2.5 D woven SiO2f/SiO2 composites using single-camera stereo-digital image correlation. Compos. Struct..

[55] Pannier Y., Foti F., Gigliotti M. (2020). High Temperature Fatigue of Carbon/Polyimide 8-harness Satin Woven Composites. Part I: Digital Image Correlation and Micro-Computed Tomography Damage Characterization. Compos. Struct..

[56] Szebényi G., Hliva V. (2019). Detection of Delamination in Polymer Composites by Digital Image Correlation—Experimental Test. Polymers.

[57] Statnik E.S., Dragu C., Besnard C., Lunt A.J.G., Salimon A.I., Maksimkin A., Korsunsky A.M. (2020). Multi-Scale Digital Image Correlation Analysis of In Situ Deformation of Open-Cell Porous Ultra-High Molecular Weight Polyethylene Foam. Polymers.

[58] Sui L., Zhong Q., Yu K., Xing F., Li P., Zhou Y. (2018). Flexural Fatigue Properties of Ultra-High Performance Engineered Cementitious Composites (UHP-ECC) Reinforced by Polymer Fibers. Polymers.

[59] Xiang-rong Y. (2010). Beam deflection measurement using one dimension digital image correlation. J. Guangzhou Univ. (Nat. Sci. Ed.).

[60] Sładek J., Ostrowska K., Kohut P., Holak K., Gąska A., Uhl T. (2013). Development of a vision based deflection measurement system and its accuracy assessment. Measurement.

[61] iMetrum. Video Gauge—How It Works. https://www.imetrum.com/video-gauge/how-it-works.

[62] El-Nashar D., Gomaa E., Abd-El-Messieh S. (2009). Study of electrical, mechanical, and nanoscale free-volume properties of NBR and EPDM rubber reinforced by bentonite or kaolin. J. Polym. Sci. Part B Polym. Phys..

[63] Özdemir T., Akbay I., Uzun H., Reyhancan I. (2016). Neutron shielding of EPDM rubber with boric acid: Mechanical, thermal properties and neutron absorption tests. Prog. Nucl. Energy.

[64] Stelescu M.D., Airinei A., Manaila E., Craciun G., Fifere N., Varganici C., Pamfil D., Doroftei F. (2018). Effects of Electron Beam Irradiation on the Mechanical, Thermal, and Surface Properties of Some EPDM/Butyl Rubber Composites. Polymers.

[65] Manaila E., Airinei A., Stelescu M.D., Sonmez M., Alexandrescu L., Craciun G., Pamfil D., Fifere N., Varganici C.-D., Doroftei F. (2020). Radiation Processing and Characterization of Some Ethylene-propylene-diene Terpolymer/Butyl (Halobutyl) Rubber/Nanosilica Composites. Polymers.

[66] Carrasco E., Passos L., Mantilla J. (2012). Structural behavior evaluation of Brazilian glulam wood sleepers when submitted to static load. Constr. Build. Mater..

[67] Derucher K., Kim U., Putcha C. (2013). Indeterminate Structural Analysis.

[68] Sedaghati R., Suleman A., Tabarrok B. (2002). Structural optimization with frequency constraints using the finite element force method. Aiaa J..

[69] Standards Australia (2010). 1720.1-2010: Timber Structures—Desing Methods.

